# Protective effects of salidroside on NAFLD rodent models by alleviating oxidative stress and inflammation: a meta-analysis and mechanism exploration

**DOI:** 10.3389/fphar.2026.1709953

**Published:** 2026-04-08

**Authors:** Shuai Li, Ran Hu, Lingyu Xu, Xin Chen, Houbo Deng

**Affiliations:** 1 College of Traditional Chinese Medicine, Changchun University of Chinese Medicine, Changchun, China; 2 The Affiliated Hospital of Changchun University of Chinese Medicine, Changchun, China

**Keywords:** inflammation, meta analysis, nonalcoholic fatty liver disease, oxidative stress, salidroside

## Abstract

**Objective:**

The purpose of this study was to systematically evaluate the therapeutic effect of salidroside on rodent NAFLD models through a meta-analysis of multiple animal experiments, and to explore its potential mechanism of anti-oxidative stress and anti-inflammation, to provide a theoretical basis for the clinical treatment of salidroside in NAFLD.

**Methods:**

A total of 12 eligible animal studies were identified by searching eight databases of Web of Science, PubMed, Embase, Cochrane Library, CNKI, Wanfang, VIP, and CBM (up to June 2025). The SYRCLE bias risk assessment tool was used to evaluate the quality of the included literature. The review used Manager 5.4 and Stata 18 software to perform a meta-analysis of the outcome indicators included in the study.

**Results:**

Meta-analysis showed salidroside significantly improved multiple outcomes: for the main outcome indicators: hepatic TG (SMD = −3.88), hepatic TC (SMD = −4.15), the NAS score (SMD = −4.79) were significantly decreased. And basic indicators, body weight (SMD = −0.75), liver weight (SMD = −1.12), and liver index (SMD = −2.24) all decreased; for lipid metabolism indicators, serum TG (SMD = −2.92), serum TC (SMD = −2.11), and LDL-C (SMD = −2.82) decreased while HDL-C (SMD = 2.34) increased, notably with dose-dependent improvements in hepatic TG and serum TC (better effect at ≥100 mg/kg/d) and rats being more sensitive to serum TG improvement; for liver function indicators, serum ALT (SMD = −3.26) and AST (SMD = −2.80) decreased, with a more pronounced effect when treatment duration was ≤4 weeks; the NAS score decreased, with a better therapeutic effect in rats than in mice; and for secondary indicators, FBG (SMD = −1.74), FSI (SMD = −1.88), HOMA-IR (SMD = −2.54), and MDA (SMD = −3.24) decreased, GSH (SMD = 3.51) and SOD (SMD = 3.96) increased, and IL-6 (SMD = −2.28), IL-1β (SMD = −1.31), and MCP-1 (SMD = −1.71) decreased. Sensitivity analysis and funnel plots indicated that the results were robust; however, a certain degree of publication bias was present.

**Conclusion:**

In rodent NAFLD models, salidroside can significantly improve the pathological state dominated by oxidative stress/inflammation. The treatment effect is affected by the treatment time, dose, type, and other factors. Its mechanism of action is mainly anti-oxidative stress and anti-inflammatory effects. Due to the differences in pathological characteristics between animal models and humans in this study, the clinical efficacy and safety of salidroside still need to be further verified by high-quality clinical studies.

**Systematic Review Registration:**

Identifier, CRD420251083205.

## 1 Introduction

Non-alcoholic fatty liver disease (NAFLD) is a progressive liver disease characterized by lipid metabolism disorders and abnormal deposition in the liver, which is induced by drugs, ethanol, genetic diseases, and other specific factors. It is often accompanied by obesity, insulin resistance, and other metabolic disorders, and is widely prevalent worldwide (Rong et al., 2022; Vujkovic et al., 2022). According to the latest statistics, the global prevalence of NAFLD is as high as 32.4%, showing a serious trend of increasing year by year, which has become a key cause of the continuous deterioration of liver disease and the occurrence and development of hepatocellular carcinoma (Riazi et al., 2022; Xue et al., 2022). The treatment of NAFLD, as outlined in the guidelines of the American Association for the Study of Liver Diseases and the European Medicines Agency (EMA), primarily advocates for intervention through exercise, weight loss, and diet control. However, there is no approved drug for this disease at present, so people turn their attention to other aspects in order to find natural compounds with high efficiency and low toxicity to treat NAFLD. This trend has gradually become mainstream in drug development and clinical medicine.

Salidroside (SDS) is the main bioactive component isolated from the roots and rhizomes of Rhodiola rosea. It belongs to phenylethanoid glycosides. It has numerous pharmacological effects, including anti-inflammatory (Zhang et al., 2021), anti-oxidative stress (Liang et al., 2024), anti-aging (Zhuang et al., 2019), immune regulation (Ye et al., 2011), and anti-tumor (Sun and Ju, 2021) properties. It has protective effects on the cardiovascular system, nervous system, liver, and kidneys. In particular, the hepatoprotective effect of SDS has been extensively explored (Wu et al., 2009; Chu et al., 2024). In the NASH rat model, SDS specifically inhibited the Nox2 and CYP2E1 enzymes involved in oxidative stress and free radical production in the liver through an antioxidant mechanism, thereby significantly reducing the degree of liver injury and abnormal lipid metabolism in NASH rats (Yang et al., 2016). In addition, several studies have confirmed that salidroside can inhibit the activity of the NLRP3 inflammasome by blocking the activation of the MAPK and NF-κB pathways (Pahl, 1999; Chang and Karin, 2001; Zhang et al., 2020), suggesting that this may be a potential target for its protective effect on liver inflammation in mice.

Therefore, in order to better understand the effect and therapeutic effect of SDS on NAFLD, and lay the foundation for the follow-up prospective clinical research, we conducted a systematic review and meta-analysis of the current available data of NAFLD rodent models treated with SDS, and verified whether its potential therapeutic mechanism mainly involves anti-oxidative stress and anti-inflammatory pathways.

## 2 Materials and methods

### 2.1 Research and design

This study has completed the prospective registration of the international system evaluation through PROSPERO, with the registration number CRD420251083205.

### 2.2 Qualification criteria

Our inclusion criteria were as follows: (1) The subjects of original studies were restricted to *in vivo* experiments on rodents; (2) The animal modeling was consistent with NAFLD; (3) Salidroside was used as the sole intervention measure (either as a pure monomer or an extract with salidroside as the sole major active ingredient), with the exclusion of any combined treatment, and the control group was either a blank control or a drug-loaded solvent; (4) The primary outcome indicators should include any one of the following:liver triglyceride (TG), liver total cholesterol (TC), the NAFLD activity score (NAS). The secondary outcome indicators should include: basic indicators (body weight, liver weight, and liver index); lipid metabolism related indicators (serum TG, serum TC, low-density lipoprotein cholesterol (LDL-C), and high-density lipoprotein cholesterol (HDL-C)); liver function related indicators (serum alanine transaminase (ALT), serum aspartate transaminase (AST)); glucose metabolism and insulin sensitivity related indicators (fasting blood glucose (FBG), fasting serum insulin (FSI), and insulin resistance (HOMA-IR)); oxidative stress related indicators (malondialdehyde (MDA), glutathione (GSH), and superoxide dismutase (SOD)); and inflammatory response related indicators (interleukin-6 (IL-6), interleukin-1β (IL-1β), and monocyte chemoattractant protein-1 (MCP-1)). All potentially relevant studies were included in the scope of full-text retrieval. In addition, if two different doses were used in the treatment group of a literature study, they would be considered as two separate studies and included in the meta-analysis, along with the control group.

### 2.3 Data selection and extraction

The data of this study were extracted by two authors. The extracted content covered general information about the study (such as the first author and year of publication), characteristics of the rodents (including species, gender, age, weight, and sample size), intervention measures (dose and treatment cycle of salidroside), modeling methods, and research results, among others. Meanwhile, a structured data extraction form was adopted. If there was any disagreement in any of the above steps, the opinion of a third person was involved for harmonization.

We searched English databases, including the Cochrane Library, PubMed, Web of Science, and Embase, as well as Chinese databases such as CNKI (China National Knowledge Infrastructure), Wanfang Data, VIP (Chinese Science and Technology Periodicals Database), and CBM (Chinese Biomedical Literature Service System). The search terms selected for this study were the combination of “salidroside” and “non-alcoholic fatty liver disease”. In the search process, we adopted a combination of MeSH terms and entry terms to conduct a comprehensive search for all references related to salidroside and non-alcoholic fatty liver disease, and supplemented the search by manually retrieving the references of eligible studies and relevant reviews. No language restrictions were applied during the search. Finally, all publications that met the inclusion criteria were imported into the literature management software EndNote 21 for systematic organization. The complete search strategy is attached in (Supplementary Material 1).

### 2.4 Risk of bias assessment

In this study, the risk of bias in all included literature was independently assessed by two evaluators. The SYRCLE risk of bias assessment tool (Hooijmans et al., 2014) is one of the most effective tools for evaluating the quality of evidence in animal studies. This tool primarily evaluated the risk of bias across the following seven aspects: selection bias, Performance bias, Detection bias, Attrition bias, Reporting bias, and Other sources of bias. A total of 10 questions were entered into Review Manager 5.4 software to assess the quality of the included studies. The judgment results were categorized as “High risk”, “Unclear risk”, or “Low risk”, and the reasons for each judgment were recorded.

### 2.5 Data analysis

In our meta-analysis, the outcome indicators of the included studies mainly involved continuous variables. Therefore, the standardized mean difference (SMD) and 95% confidence interval (95% CI) were used, and the results were summarized in RevMan 5.4.1 software to generate forest plots. We further used I^2^ and p-values to assess the heterogeneity among the included studies. If I^2^ > 50% or p < α (α = 0.1), it indicates a high level of heterogeneity; when I^2^ > 75%, there is high heterogeneity in effect sizes among the studies. In such cases, a random-effects model was used to pool the data (Higgins et al., 2003), and a one-by-one exclusion method was applied to each study to observe changes in heterogeneity and identify the causes of heterogeneity. In addition, for data with high heterogeneity among outcome indicators, we conducted subgroup analyses, using treatment duration, dosage, and rodent species as the bases for subgrouping to observe changes in heterogeneity and study the impact of different factors on outcome indicators. Meanwhile, we used Stata 18 software to perform sensitivity analyses on effect sizes and analyzed publication bias by drawing funnel plots and conducting Egger’s tests. When the p-value >0.05, it suggests no significant publication bias; if the p-value ≤0.05, it indicates the presence of publication bias.

## 3 Results

**FIGURE 1:**
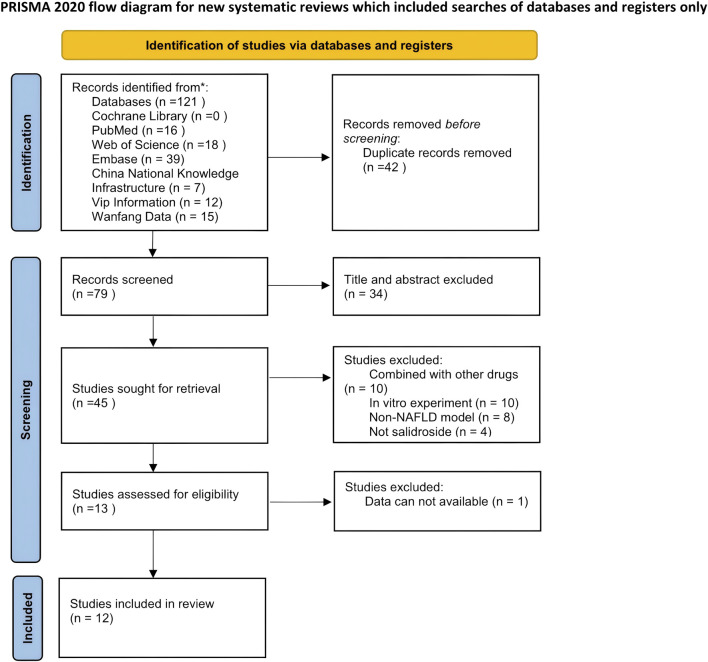
Flow diagram for systematic literature search.

**FIGURE 2:**
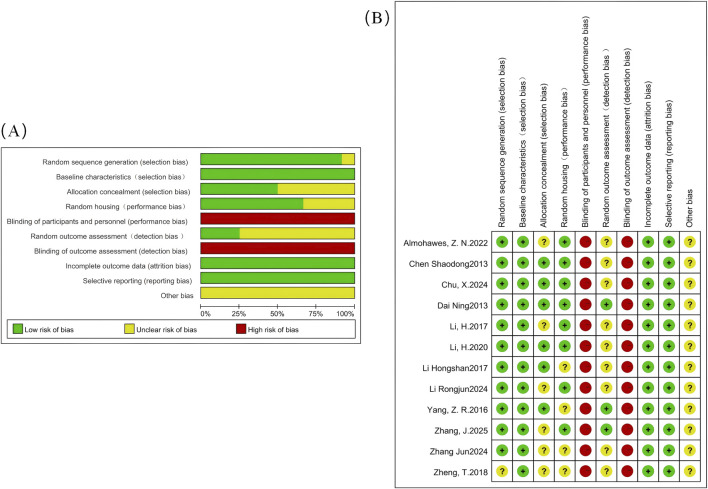
The risk of bias assessment. **(A)** Risk of bias graph; **(B)** Risk of bias summary.

According to the systematic search strategy, the total number of initially included literature in this study was 121. After duplicate removal, the number of valid literature was reduced to 79. Subsequently, an initial screening of titles and abstracts was conducted, and 34 studies were excluded based on criteria such as the relevance of research objects, intervention measures, and study types. All of these excluded studies were review articles, leaving 45 studies to enter the full-text in-depth evaluation stage. After content review, 32 additional studies that did not meet the inclusion criteria were excluded. A total of 13 studies ultimately met the inclusion criteria; however, one of them could not be included in the subsequent meta-analysis due to the unavailability of data (Hu et al., 2021). Therefore, 12 studies ultimately met the inclusion criteria and were included in this systematic review; the specific retrieval process is illustrated in Figure 1. The main characteristics of all included studies are summarized in Table 1. The overall risk of bias of the included studies is shown in Figure 2.

**TABLE 1:** Characteristics of studies included.

Study ID	Dosage (mg/kg/d)	Therapeutic time	Rats/Mice	Modeling method	Sample	Outcomes
Chu et al. (2024)	24	4 Weeks	Mice	MCD	30 (15:15)	1,2,3,4,5,6,7,10,11,12,19
Almohawes et al. (2022)	300	12 Weeks	Rats	HFD	16 (8:8)	1,2,3,4,5,6,7,8,9,13,14,15,16,17,18,19
Li et al. (2020)	20	4 Weeks	Mice	HFD	16 (8:8)	1,2,3,4,5,6,7,8,9,10,11,12,21
Li et al. (2017)	4.33	6 Weeks	Rats	HFD	16 (8:8)	1,2,4,5,10,11,13,14,15
Yang et al. (2016)	150/300	6 Weeks	Rats	HFHC	20 (10:10)	2,3,4,5,10,11,12,16,17,18
Zhang et al. (2025)	25/50	4 Weeks	Mice	CDAHFD	16 (8:8)	2,3,4,10,11,12,19,20,21
Zheng et al. (2018)	100	8 Weeks	Mice	HFD	14 (7:7)	1,2,3,4,5,6,7,8,9,10,11,12,13,14,15,16,18,20,21
Shao-dong et al. (2013)	100	4 Weeks	Rats	HFD	12 (6:6)	1,2,3,4,5,6,7,8,9,10,11,12,19
Ning et al. (2013)	300	6 Weeks	Rats	HFD	20 (10:10)	2,3,4,5,10,11,12,16,17,18
Hong-shan et al. (2017)	43.3	6 Weeks	Rats	HFD	16 (8:8)	1,2,3,4,5,10,11,12,16,18
Jun et al. (2024)	50	6 Weeks	Mice	HFD	16 (8:8)	2,3,4,5,10,11,12,19,20,21
Rongjun et al. (2024)	100	8 Weeks	Mice	HFD	12 (6:6)	1,2,3,4,5,10,11,12,13,14,15,16,17,18,19,20

1: body weight; 2: liver weight; 3: liver index; 4: Liver TG; 5: Liver TC; 6: serum TG; 7: serum TC; 8: LDL-C; 9: HDL-C; 10: serum ALT; 11: serum AST; 12: NAS; 13: FBG; 14: FSI; 15: HOMA-IR; 16: MDA; 17: GSH; 18: SOD; 19: IL-6; 20: IL-1β; 21: MCP-1. MCD: methionine and choline-deficient diet; HFD: High-fat diet; HFHC: high fat and high cholesterol diet; CDAHFD: Choline-deficient L-amino acid high fat diet.

### 3.1 Main outcome indicators

#### 3.1.1 Liver TG and TC

**FIGURE 3:**
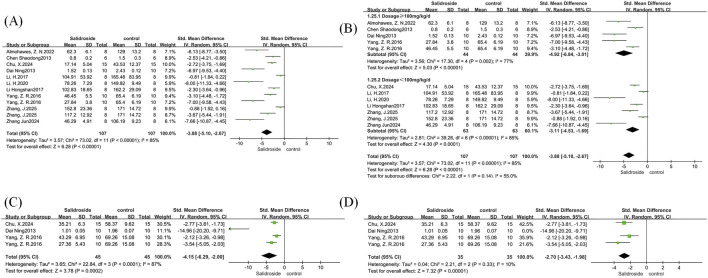
Liver TG and TC forest maps. Note: **(A)** Liver TG forest map; **(B)** Liver TG subgroup forest map; **(C)** Liver TC forest map; **(D)** Liver TC subgroup forest map.

We conducted a meta-analysis of liver TG and TC. A total of 12 studies (Shao-dong et al., 2013; Ning et al., 2013; Yang et al., 2016; Li et al., 2017; Hong-shan et al., 2017; Li et al., 2020; Almohawes et al., 2022; Chu et al., 2024; Jun et al., 2024; Zhang et al., 2025) were included in the analysis of liver TG, and four studies (Ning et al., 2013; Yang et al., 2016; Chu et al., 2024) were involved in the analysis of liver TC, with all results reported as SMD. Both analyses showed high heterogeneity: liver TG (I^2^ = 85%, p < 0.00001) and liver TC (I^2^ = 87%, p < 0.0001); therefore, a random-effects model was used for both. The analysis results showed that the salidroside treatment group had significantly better effects on liver TG (SMD = −3.88, 95% CI = −5.10 to −2.67, p < 0.00001) (Figure 3A) and liver TC (SMD = −4.15, 95% CI = −6.29 to −2.00, p = 0.0002) (Figure 3C) compared with the control group, with significant reductions in both indicators. We then performed subgroup analyses for both indicators. In the liver TG group, with salidroside dosage as the grouping basis, the results showed that a dosage of ≥100 mg/kg/d had a better therapeutic effect (Figure 3B). In the liver TC group, due to the small number of included studies, we used the one-by-one exclusion method. After excluding one study (Ning et al., 2013), the heterogeneity decreased significantly (I^2^ = 10%, p = 0.33) (Figure 3D). A subsequent thorough review of the excluded literature revealed that differences in measurement methods may have contributed to the high heterogeneity.

#### 3.1.2 NAFLD activity score (NAS)

**FIGURE 4:**
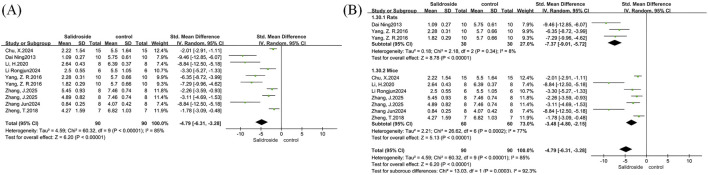
NAS forest maps. Note: **(A)** NAS forest map; **(B)** NAS subgroup forest map.

The meta-analysis of NAS was conducted on 10 studies (Ning et al., 2013; Yang et al., 2016; Zheng et al., 2018; Li et al., 2020; Chu et al., 2024; Jun et al., 2024; Rongjun et al., 2024; Zhang et al., 2025), with results reported as SMD. The analysis involved a total of 180 samples, and compared with the control group, the NAS in the salidroside treatment group decreased significantly (SMD = −4.79, 95% CI = −6.31 to −3.28, p < 0.00001) (Figure 4A), indicating a remarkable therapeutic effect. Significant heterogeneity was observed among the studies (I^2^ = 85%, p < 0.00001); therefore, a random-effects model was adopted. We used rodent species as the basis for subgroup analysis. The results showed that under salidroside treatment, the NAS of the rat group decreased significantly. Compared with the mouse group, the rat group achieved a better therapeutic effect, and heterogeneity was significantly reduced (I^2^ = 8%, p = 0.34) (Figure 4B). This suggested that the source of heterogeneity might be the difference in rodent species included in the studies.

### 3.2 Secondary outcome indicators

#### 3.2.1 Basic indicators

**FIGURE 5:**
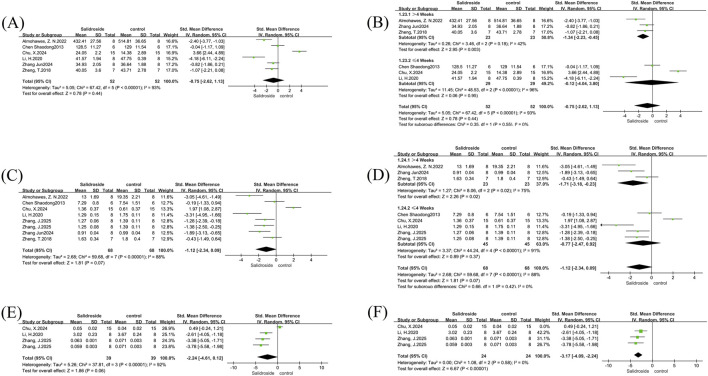
Basic indicators forest maps. Note: **(A)** body weight forest map; **(B)** body weight subgroup forest map; **(C)** liver weight forest map; **(D)** liver weight subgroup forest map; **(E)** liver index forest map; **(F)** liver index subgroup forest map.

A meta-analysis of body weight was performed on six studies (Shao-dong et al., 2013; Zheng et al., 2018; Li et al., 2020; Almohawes et al., 2022; Chu et al., 2024; Jun et al., 2024), with results reported as SMD. The analysis involved a total of 104 samples, and there was no significant difference in body weight change between the treatment group and the control group (SMD = −0.75, 95% CI = −2.62 to 1.13, p = 0.44). Significant heterogeneity was observed among the studies (I^2^ = 93%, p < 0.00001) (Figure 5A); therefore, a random-effects model was adopted. We used treatment duration as the basis for subgroup analysis to observe the impact of different treatment durations of salidroside on body weight changes. The results showed that when the treatment duration was ≤4 weeks, no significant difference was found between the salidroside treatment group and the control group; however, when the treatment duration exceeded 4 weeks, the body weight in the salidroside treatment group decreased significantly, suggesting that long-term administration of salidroside has a better weight-loss effect in NAFLD rodents (Figure 5B).

A meta-analysis of liver weight was conducted on eight studies (Shao-dong et al., 2013; Zheng et al., 2018; Li et al., 2020; Almohawes et al., 2022; Chu et al., 2024; Jun et al., 2024; Zhang et al., 2025), with results reported as SMD. The analysis included a total of 136 samples, and there was no statistically significant difference in the salidroside treatment group compared with the control group (SMD = −1.12, 95% CI = −2.34 to 0.09, p = 0.07). Significant heterogeneity was observed among the studies (I^2^ = 88%, p < 0.00001) (Figure 5C); therefore, a random-effects model was used. In the subgroup analysis based on different salidroside treatment durations, we found that when the treatment duration was ≤4 weeks, no significant difference was observed in this indicator. However, long-term salidroside treatment (>4 weeks) showed a significant therapeutic effect on reducing liver weight (Figure 5D).

A meta-analysis of liver index was performed on four studies (Li et al., 2020; Chu et al., 2024; Zhang et al., 2025), with results reported as SMD. The analysis involved a total of 78 samples, and there was no significant difference between the salidroside treatment group and the control group (SMD = −2.24, 95% CI = −4.61 to 0.12, p = 0.06). Significant heterogeneity was observed among the studies (I^2^ = 92%, p < 0.00001) (Figure 5E); therefore, a random-effects model was adopted, and a one-by-one exclusion method was employed. After excluding one study (Chu et al., 2024), we found that the heterogeneity in this analysis decreased significantly (I^2^ = 0%, p = 0.58), and the effect of the salidroside treatment group was significantly better than that of the control group (Figure 5F). Subsequently, a thorough review of the excluded literature revealed that the possible cause of heterogeneity was the difference in modeling methods. This study employed an MCD diet for induction. Due to the lack of methionine in the modeled animals, their body weight was significantly reduced, which had a substantial impact on the liver index level (Jahn et al., 2019).

#### 3.2.2 Lipid metabolism related indicators

**FIGURE 6:**
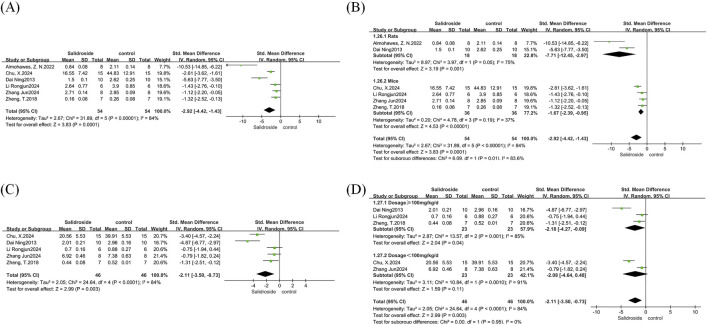
serum TG and TC forest maps. Note: **(A)** serum TG forest map; **(B)** serum TG subgroup forest map; **(C)** serum TC forest map; **(D)** serum TC subgroup forest map.

We conducted a meta-analysis of serum TG and TC. A total of six studies (Ning et al., 2013; Zheng et al., 2018; Almohawes et al., 2022; Chu et al., 2024; Jun et al., 2024; Rongjun et al., 2024) were included in the analysis of serum TG, and five studies (Ning et al., 2013; Zheng et al., 2018; Chu et al., 2024; Jun et al., 2024; Rongjun et al., 2024) were involved in the analysis of serum TC, with all results reported as SMD. Both analyses showed high heterogeneity: serum TG (I^2^ = 84%, p < 0.00001) and serum TC (I^2^ = 84%, p < 0.0001); therefore, a random-effects model was used for both. The analysis results showed that the salidroside treatment group had significantly better effects on serum TG (SMD = −2.92, 95% CI = −4.42 to −1.43, p = 0.0001) (Figure 6A) and serum TC (SMD = −2.11, 95% CI = −3.50 to −0.73, p = 0.003) (Figure 6C) compared with the control group, with significant reductions in both indicators. We then performed subgroup analyses for both indicators. In the serum TG group, with rodent species as the grouping basis, the results showed that the rat group was more sensitive to salidroside and achieved better therapeutic effects (Figure 6B). In the serum TC group, with salidroside dosage as the grouping basis, the results showed that when the dosage was <100 mg/kg/d, there was no difference between the treatment group and the control group; however, when the dosage was ≥100 mg/kg/d, the serum TC level in the salidroside treatment group decreased significantly, showing a statistical difference (Figure 6D).

**FIGURE 7:**
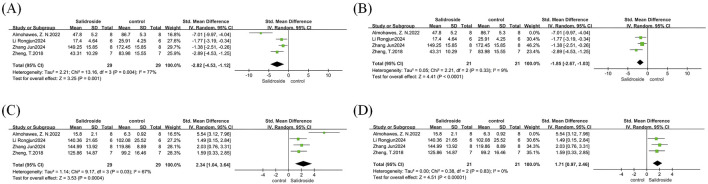
LDL-C and HDL-C forest maps. Note: **(A)** LDL-C forest map; **(B)** LDL-C subgroup forest map; **(C)** HDL-C forest map; **(D)** HDL-C subgroup forest map.

We conducted a meta-analysis of LDL-C and HDL-C, involving four studies (Zheng et al., 2018; Almohawes et al., 2022; Jun et al., 2024; Rongjun et al., 2024) that analyzed both indicators, and all results were reported as SMD. Both analyses showed high heterogeneity: LDL-C (I^2^ = 77%, p = 0.004) and HDL-C (I^2^ = 67%, p = 0.03); therefore, a random-effects model was used for both. The analysis results showed that the salidroside treatment group could effectively regulate the levels of LDL-C (SMD = −2.82, 95% CI = −4.53 to −1.12, p = 0.001) (Figure 7A) and HDL-C (SMD = 2.34, 95% CI = 1.04 to 3.64, p = 0.0004) (Figure 7C), which was statistically significant. We applied the one-by-one exclusion method to both groups of analyses and found that after excluding one study (Almohawes et al., 2022), the heterogeneity of both groups decreased significantly: the LDL-C group (I^2^ = 9%, p = 0.33) (Figure 7B) and the HDL-C group (I^2^ = 0%, p = 0.83) (Figure 7D). A subsequent thorough review of the excluded literature suggested that the heterogeneity might be related to the dosage of salidroside and the rodent species. The excluded literature used a salidroside dosage of 300 mg/kg/d and Wistar rats, while the other three studies used a dosage of ≤100 mg/kg/d and mice; this difference might have led to the significant change in heterogeneity.

#### 3.2.3 Liver function related indicators

**FIGURE 8:**
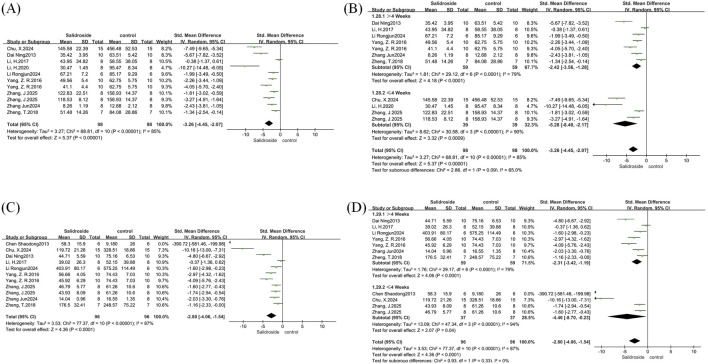
serum ALT and AST forest maps. Note: **(A)** serum ALT forest map; **(B)** serum ALT subgroup forest map; **(C)** serum AST forest map; **(D)** serum AST subgroup forest map.

We conducted a meta-analysis of serum ALT and AST. A total of 11 studies (Ning et al., 2013; Yang et al., 2016; Li et al., 2017; Zheng et al., 2018; Li et al., 2020; Chu et al., 2024; Jun et al., 2024; Rongjun et al., 2024; Zhang et al., 2025) were included in the analysis of serum ALT, and 11 studies (Ning et al., 2013; Yang et al., 2016; Li et al., 2017; Zheng et al., 2018; Chu et al., 2024; Jun et al., 2024; Rongjun et al., 2024; Zhang et al., 2025) were involved in the analysis of serum AST, with all results reported as SMD. Both analyses showed high heterogeneity: serum ALT (I^2^ = 85%, p < 0.00001) and serum AST (I^2^ = 87%, p < 0.00001); therefore, a random-effects model was used for both. The analysis results showed that the salidroside treatment group could significantly reduce the levels of serum ALT (SMD = −3.26, 95% CI = −4.45 to −2.07, p < 0.00001) (Figure 8A) and serum AST (SMD = −2.80, 95% CI = −4.06 to −1.54, p < 0.0001) (Figure 8C). Its therapeutic effect was significantly better than that of the control group, which was statistically significant. We used the treatment duration of salidroside as the basis for subgroup analysis. Interestingly, it was found that when the treatment duration was ≤4 weeks, the therapeutic effects on serum ALT and AST were better than those of long-term treatment (Figures 8B,D). However, the specific reason has not been verified by studies so far; we speculate that it may be related to the body’s metabolic regulation or other confounding factors.

#### 3.2.4 Glucose metabolism and insulin sensitivity related indicators

**FIGURE 9:**
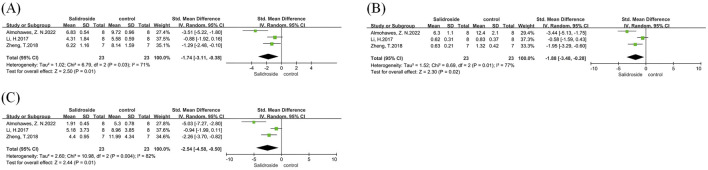
FBG, FSI, and HOMA-IR forest maps. Note: **(A)** FBG forest map; **(B)** FSI forest map; **(C)** HOMA-IR forest map.

We conducted a meta-analysis of FBG, FSI, and HOMA-IR, involving three studies (Li et al., 2017; Zheng et al., 2018; Almohawes et al., 2022) that analyzed all three indicators. All results were reported as SMD. All three analyses showed high heterogeneity: FBG (I^2^ = 71%, p = 0.03), FSI (I^2^ = 77%, p = 0.01), and HOMA-IR (I^2^ = 82%, p = 0.004). Therefore, a random-effects model was used for all of them. The analysis results showed that compared with the control group, the FBG level in the salidroside treatment group decreased significantly (SMD = −1.74, 95% CI = −3.11 to −0.38, p = 0.01) (Figure 9A), the FSI level reduced significantly (SMD = −1.88, 95% CI = −3.48 to −0.28, p = 0.02) (Figure 9B), and the HOMA-IR level decreased significantly (SMD = −2.54, 95% CI = −4.58 to −0.50, p = 0.01) (Figure 9C). Due to the small number of literature studies on these indicators, no subgroup discussion was conducted for them.

#### 3.2.5 Oxidative stress related indicators

**FIGURE 10:**
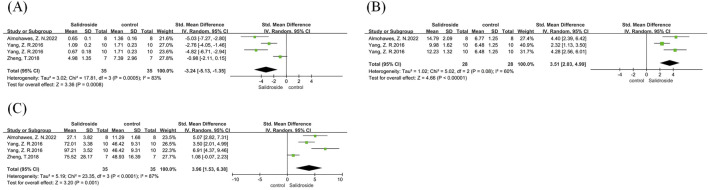
MDA, GSH, and SOD forest maps. Note: **(A)** MDA forest map; **(B)** GSH forest map; **(C)** SOD forest map.

We conducted a meta-analysis of MDA, GSH, and SOD. A total of four studies (Yang et al., 2016; Zheng et al., 2018; Almohawes et al., 2022) were involved in the analysis of MDA and SOD, and three studies (Yang et al., 2016; Almohawes et al., 2022) were included in the analysis of GSH, with all results reported as SMD. All three analyses showed high heterogeneity: MDA (I^2^ = 83%, p = 0.0005), GSH (I^2^ = 60%, p = 0.08), and SOD (I^2^ = 87%, p < 0.0001). Therefore, a random-effects model was used for all of them. The analysis results showed that compared with the control group, the MDA level in the salidroside treatment group decreased significantly (SMD = −3.24, 95% CI = −5.13 to −1.35, p = 0.0008) (Figure 10A), the GSH content increased significantly (SMD = 3.51, 95% CI = 2.03 to 4.99, p < 0.00001) (Figure 10B), and the SOD level rose significantly (SMD = 3.96, 95% CI = 1.53 to 6.38, p = 0.001) (Figure 10C).

#### 3.2.6 Inflammatory response related indicators

**FIGURE 11:**
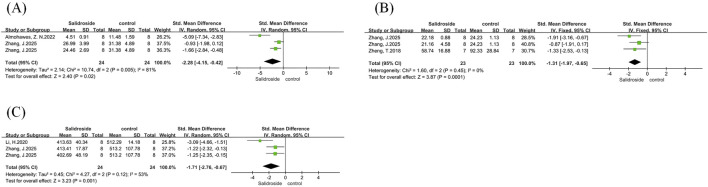
IL-6, IL-1β, and MCP-1 forest maps. Note: **(A)** IL-6 forest map; **(B)** IL-1β forest map; **(C)** MCP-1 forest map.

We conducted a meta-analysis of IL-6, IL-1β, and MCP-1. A total of three studies (Almohawes et al., 2022; Zhang et al., 2025) were involved in the analysis of IL-6, three studies (Zheng et al., 2018; Zhang et al., 2025) in the analysis of IL-1β, and three studies (Li et al., 2020; Zhang et al., 2025) in the analysis of MCP-1, with all results reported as SMD. Due to the high heterogeneity observed in the analyses of IL-6 (I^2^ = 81%, p = 0.0005) and MCP-1 (I^2^ = 53%, p = 0.12), a random-effects model was adopted for these two indicators. In contrast, IL-1β showed low heterogeneity (I^2^ = 0%, p = 0.445), so a fixed-effects model was used for its analysis. The results indicated that compared with the control group, the salidroside treatment group had a significant decrease in IL-6 level (SMD = −2.28, 95%CI = −4.15 to −0.42, p = 0.02) (Figure 11A), a significant reduction in IL-1β content (SMD = −1.31, 95% CI = −1.97 to −0.65, p = 0.0001) (Figure 11B), and a significant decrease in MCP-1 level (SMD = −1.71, 95% CI = −2.76 to −0.67, p = 0.001) (Figure 11C).

### 3.3 Publication bias

**FIGURE 12:**
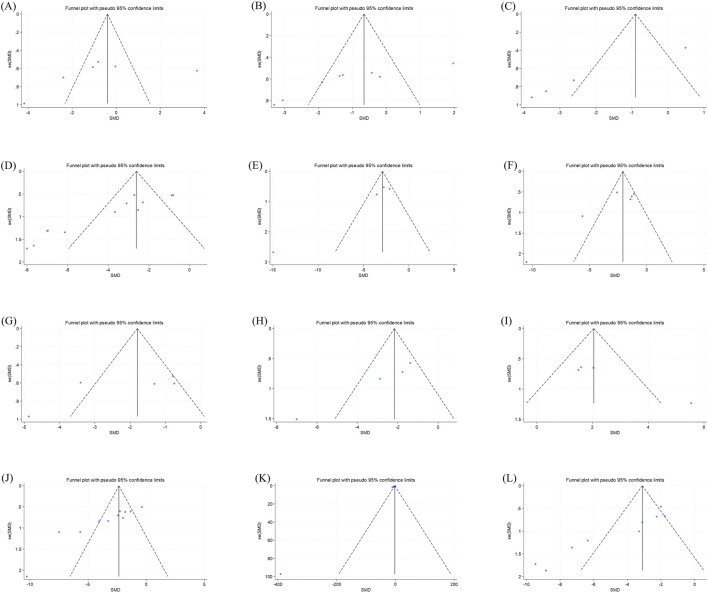
Primary outcome indicators funnel plots. Note: **(A)** body weight funnel plot; **(B)** liver weight funnel plot; **(C)** liver index funnel plot; **(D)** Liver TG funnel plot; **(E)** Liver TC funnel plot; **(F)** serum TG funnel plot; **(G)** serum TC funnel plot; **(H)** LDL-C funnel plot; **(I)** HDL-C funnel plot; **(J)** serum ALT funnel plot; **(K)** serum AST funnel plot; **(L)** NAS funnel plot.

We assessed the publication bias of the primary outcome indicators using funnel plots and Egger’s test. The funnel plots for body weight and serum TC showed a symmetric distribution, and the results of Egger’s test indicated a low degree of publication bias. In contrast, the funnel plots for the other primary outcome indicators showed an asymmetric distribution, and the results of Egger’s test suggested significant publication bias. The main reason for this was that most of the animal experiment studies included in this research were published with positive results. Details are shown in Table 2 and Figure 12.

**TABLE 2:** Egger test.

Number	Main outcome indicators	t	p	95% Conf. interval	
1	Liver TG	−7.34	0.000	−7.76076	−4.146629
2	Liver TC	−5.28	0.034	−10.31679	−1.054683
3	NAS	−8.57	0.000	−6.832581	−3.934053
4	Body weight	−0.94	0.399	−31.61147	15.57769
5	liver weight	−4.97	0.003	−20.46111	−6.95364
6	liver index	−27.63	0.001	−9.339513	−6.822824
7	serum TG	−2.99	0.041	−10.34877	−0.3756638
8	serum TC	−1.77	0.175	−24.51958	7.012963
9	LDL-C	−8.46	0.014	−9.026348	−2.939404
10	HDL-C	4.94	0.039	0.8377766	12.13232
11	serum ALT	−7.24	0.000	−10.10565	−5.29612
12	serum AST	−5.81	0.000	−8.697096	−3.820242

### 3.4 Sensitivity analysis

**FIGURE 13:**
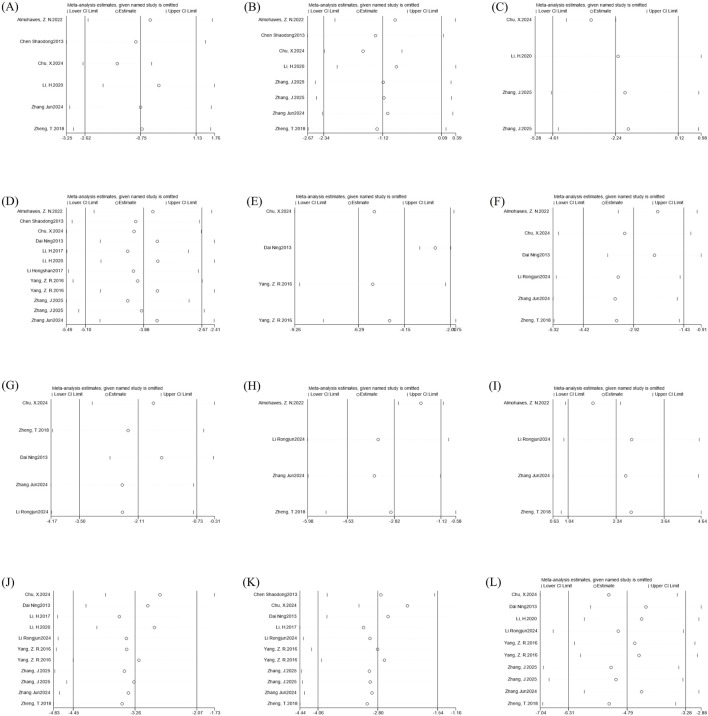
Sensitivity analysis of primary outcome indicators. Note: **(A)** Sensitivity Analysis of body weight; **(B)** Sensitivity Analysis of liver weight; **(C)** Sensitivity Analysis of liver index; **(D)** Sensitivity Analysis of Liver TG; **(E)** Sensitivity Analysis of Liver TC; **(F)** Sensitivity Analysis of serum TG; **(G)** Sensitivity Analysis of serum TC; **(H)** Sensitivity Analysis of LDL-C; **(I)** Sensitivity Analysis of HDL-C; **(J)** Sensitivity Analysis of serum ALT; **(K)** Sensitivity Analysis of serum AST; **(L)** Sensitivity Analysis of NAS.

After excluding one study at a time and re-conducting the meta-analysis, there was no significant difference between the pooled effect sizes of each outcome indicator and the results of the full-sample analysis (Figure 13). The sensitivity analysis showed that excluding any single study did not significantly change the direction or magnitude of the treatment effect.

### 3.5 Mechanism research

**FIGURE 14:**
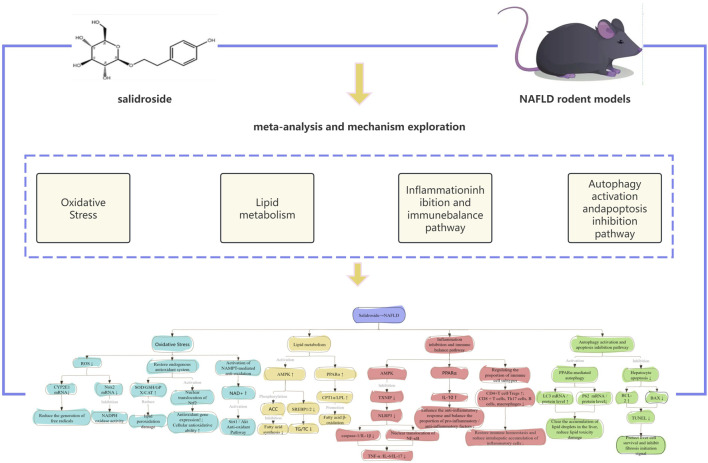
Mechanism map of salidroside in the treatment of NAFLD. Note: The blue module represents the oxidative stress regulation pathway, the yellow module represents the lipid metabolism reprogramming pathway, the red module represents the inflammation inhibition and immune balance pathway, and the green module represents the autophagy activation and apoptosis inhibition pathway. The ' ↓ ' between the two boxes in the article indicates that there is downstream regulation; the ' ↑ ' and ' ↓ ' after the protein indicate changes in the content of the regulated protein or gene after treatment. The description appearing on the downregulation arrow is the way of regulation.

Based on the analysis of the studies included in this research, we found that salidroside can treat non-alcoholic fatty liver disease (NAFLD) through multiple pathways and routes. We illustrated the specific mechanisms in the form of a flow chart, which is shown in Figure 14.

## 4 Discussion

In this study, we included 12 pieces of literature, involving a total of 14 animal experimental studies, to evaluate various indicators of salidroside in the treatment of NAFLD. First, in terms of basic indicators, there were no significant differences between the salidroside treatment group and the control group in the effects on body weight, liver weight, and liver index. Notably, when the treatment duration exceeded 4 weeks, the results for body weight and liver weight were statistically significant, suggesting that salidroside may require a specific treatment cycle to exert a stable therapeutic effect. In evaluating the treatment effect on the liver index, differences in modeling methods also significantly impacted the results. Second, regarding lipid metabolism related indicators, salidroside showed an apparent improving effect on liver TG, liver TC, serum TG, serum TC, LDL-C, and HDL-C. During the subgroup analysis, we found that salidroside exerted its therapeutic effect on liver TG and serum TC in a dose-dependent manner. In addition, compared with the mouse group, the rat group achieved a better effect on serum TG. In terms of liver function related indicators, the therapeutic effect of the salidroside treatment group was significantly better than that of the control group. Interestingly, when the treatment duration was prolonged, its therapeutic effect decreased slightly. We speculate that this may be related to metabolic regulation in the mouse body or other confounding factors; however, no relevant studies have yet verified this. Furthermore, the salidroside treatment group also showed apparent improvement in NAS score, glucose metabolism, and insulin sensitivity related indicators, as well as oxidative stress related indicators. This further confirms the therapeutic effectiveness of salidroside in the treatment of NAFLD in rodents.

Subsequently, we conducted a publication bias detection on all the included literature. Given that most current animal experimental studies tend to publish positive results, and factors such as differences in modeling methods, geographical locations, species, and measurement methods exist, the potential impact of publication bias cannot be completely ruled out. This has also caused significant heterogeneity in most outcome indicators of this study. If more similar studies are published in the future, correction methods can be employed to validate further the stability of the conclusions drawn from this study. In addition, the results of our sensitivity analysis showed that when any single study was excluded and the analysis was re-conducted, all the obtained results fell within the confidence interval, indicating that the analysis results are robust.

In this meta-analysis, the mechanisms of salidroside in treating NAFLD were generally categorized into four types: the antioxidant stress pathway, anti-inflammatory pathway, lipid metabolism pathway, and autophagy pathway. Among these, the antioxidant stress pathway and anti-inflammatory pathway were the main ones.

Oxidative stress is closely associated with NAFLD. Antioxidants can improve the formation and progression of NAFLD. CYP2E1 (cytochrome P450 2E1) is a major drug/toxin-metabolizing enzyme in liver microsomes, belonging to the cytochrome P450 superfamily. It is mainly distributed in the endoplasmic reticulum of hepatocytes, with a small amount present in mitochondria. Its core physiological function is to participate in the oxidative metabolism of aliphatic and aromatic compounds (such as alcohol, fatty acids, and drugs); however, the metabolic process is accompanied by the generation of reactive oxygen species (ROS), which is the core entry point for its association with the pathological mechanism of NAFLD (Lee et al., 2011). Oxidative stress mediated by CYP2E1 further activates intrahepatic inflammatory signals. An increase in the expression level of CYP2E1 can induce the generation of more free electrons, and this process is coupled with the enzymatic conversion of reduced nicotinamide adenine dinucleotide phosphate (NADPH) to oxidized nicotinamide adenine dinucleotide phosphate (NADP^+^) via Nox2 and/or Nox4. Due to the incomplete reaction coupling in the reaction cycle of CYP2E1, the catalytic process is accompanied by the generation of ROS. At the same time, Nox2 and Nox4 can also promote the recycling of NADP^+^, thereby facilitating the production of superoxide anions and peroxides; these peroxides and ROS can further generate active substances with stronger toxicity (Shah et al., 2013). During the treatment process, salidroside can restore the endogenous antioxidant system. It mainly achieves this by increasing the activities of SOD/GSH/GPX/CAT, alleviating lipid peroxidation damage, activating the nuclear translocation of Nrf2, upregulating the expression of antioxidant genes, enhancing cellular antioxidant capacity, reducing the level of hepatic MDA, and protecting hepatocytes from oxidative damage (Zhu and Yu, 2024).

The accumulation of free fatty acids (FFA) caused by lipid metabolism disorders is the primary link initiating the progression of non-alcoholic fatty liver disease (NAFLD). Abnormal accumulation of fatty acids promotes adipogenesis, and this change may contribute to the progression of NAFLD (Qu et al., 2022). In recent years, the use of salidroside to improve lipid metabolism has shown a positive effect in the treatment of NAFLD. Salidroside can alleviate lipid accumulation in hepatocytes and the liver induced by palmitic acid/oleic acid (PO) or high-fat/high-cholesterol (HFHC) stimulation through the AMPK signaling pathway, while exerting an inhibitory effect on liver injury. Additionally, relevant studies have confirmed that the composite formula of salidroside and curcumin has a significant inhibitory effect on lipid accumulation in the livers of NAFLD model rats (Li, 2018). A growing body of research evidence shows that salidroside can also regulate other targets closely related to lipid metabolism, including Micro-R-370 and insulin resistance (IR), and reduce lipid accumulation through this regulatory mechanism. The role of microRNAs (miRNAs) in lipid metabolism disorders has been widely explored; among them, Micro-R-370 has been confirmed to regulate mRNAs related to SREBP-1c, FAS, DGAT2, and Acc1, and promote hepatic adipogenesis (Iliopoulos et al., 2010). In addition, this meta-analysis also revealed that salidroside can upregulate the expression level of PPARα, thereby promoting the expression of carnitine palmitoyltransferase 1α (CPT1α) and lipoprotein lipase (LPL), which in turn accelerates fatty acid β-oxidation. At the same time, it can inhibit fatty acid synthase (FAS), reduce the activity of key enzymes in lipid synthesis, and finally achieve the effects of reducing the levels of intrahepatic triglycerides (TG), total cholesterol (TC), and free fatty acids (FFA), while improving the serum lipid profile and alleviating the degree of hepatic steatosis.

Thioredoxin-interacting protein (TXNIP) is an endogenous negative regulator of intracellular glutathione reductase, which functions by inhibiting the antioxidant activity of this enzyme. Meanwhile, TXNIP can dissociate from thioredoxin (TRX) and interact with NOD-like receptor pyrin domain-containing protein 3 (NLRP3), thereby activating the inflammasome (Zhou et al., 2010). Inflammasomes are a class of cytoplasmic multiprotein complexes composed of various NOD-like receptors (NLRs) and PYHIN family proteins, whose main members include NLRP1, NLRP3, NLRC4, and AIM2 (Henao-Mejia et al., 2012). As molecular sensors capable of detecting endogenous/exogenous pathogen-associated molecular patterns (PAMPs) or damage-associated molecular patterns (DAMPs), inflammasomes not only regulate the production of interleukin-1β (IL-1β) and interleukin-18 (IL-18) but also indirectly control the secretion of pro-inflammatory cytokines such as tumor necrosis factor-α (TNF-α), interleukin-6 (IL-6), and interleukin-17 (IL-17) by affecting the expression level of nuclear factor κB (NF-κB) (Henao-Mejia et al., 2012; Feng et al., 2019). In addition, salidroside can activate the peroxisome proliferator-activated receptor α (PPARα) signaling pathway and exert anti-inflammatory effects through the PPARα-mediated transcriptional regulation mechanism. This component can significantly upregulate the expression and secretion of the anti-inflammatory cytokine interleukin-10 (IL-10). IL-10 can enhance the body’s anti-inflammatory response capacity by inhibiting macrophage activation and reducing the release of pro-inflammatory factors. Beyond regulating the inflammatory response, studies have found that salidroside can also specifically regulate the population ratio of immune cell subtypes: in liver and spleen tissues, it can increase the relative proportion of CD4^+^ T lymphocytes and regulatory T cells (Tregs), while decreasing the proportion of CD8^+^ T lymphocytes, T helper 17 cells (Th17), B lymphocytes, and macrophages. Furthermore, salidroside can reduce the infiltration of natural killer cells (NK cells) into liver tissue, ultimately restoring immune homeostasis and reducing the accumulation of inflammatory cells in the liver.

In the disease progression of NAFLD, lipotoxic damage and excessive apoptosis of hepatocytes are the core pathological links driving the development of liver fibrosis. Salidroside can maintain hepatocyte survival and function through two dual mechanisms: “activating autophagy to clear lipotoxicity” and “inhibiting apoptosis to protect cells”, ultimately achieving protection of the structural integrity of liver tissue (Chu et al., 2024). On the one hand, salidroside can activate the peroxisome proliferator-activated receptor α (PPARα) signaling pathway. By regulating the expression of autophagy-related genes, it initiates the autophagic process, significantly upregulates the mRNA and protein levels of microtubule-associated protein one light chain 3 (LC3), and promotes the conversion of LC3-I to LC3-II (a marker protein of autophagosomal membrane). Meanwhile, it downregulates the mRNA and protein expression of the autophagic substrate protein p62 (SQSTM1) to ensure the normal flow of autophagy. Further increases the formation of autophagosomes, which specifically encapsulate the lipid droplets accumulated in hepatocytes to form “lipophagosomes”. After fusing with lysosomes to degrade lipid droplets, lipophagosomes reduce the lipotoxic damage caused by free fatty acids and their metabolic intermediates, and avoid mitochondrial dysfunction and endoplasmic reticulum stress induced by lipotoxicity (Gordy and He, 2012).

On the other hand, salidroside can inhibit excessive apoptosis of hepatocytes by regulating the balance of apoptosis-related proteins (Ho et al., 2021; Wang et al., 2022). Specifically, it significantly upregulates the anti-apoptotic protein B-cell lymphoma-2 (BCL-2) to stabilize the mitochondrial membrane potential and block the release of cytochrome c; simultaneously, it downregulates the pro-apoptotic protein BCL-2-associated X protein (BAX) to inhibit the initiation of the apoptotic cascade. Ultimately, this reduces the number of TUNEL-positive cells (a marker of DNA fragmentation), which not only decreases hepatocyte loss but also prevents damage-associated molecular patterns (DAMPs) released by apoptotic cells from activating intrahepatic immune cells and the secretion of pro-fibrotic factors (such as TGF-β1), thereby blocking the initiating signals of fibrosis. Under the combined effect of these two mechanisms, the reduced lipotoxicity caused by autophagy activation decreases the sensitivity of hepatocytes to apoptotic signals. At the same time, the inhibition of apoptosis provides sufficient time for autophagy-mediated damage repair. Together, they significantly reduce the level of hydroxyproline (HYP) (an indicator of collagen accumulation) in liver tissue, ultimately maintaining the normal morphology and function of hepatocytes, reducing the destruction of hepatic lobule structure, and achieving the protection of the structural integrity of liver tissue.

Collectively, the hepatoprotective effects of salidroside against NAFLD in rodent models are not mediated by four isolated independent pathways (antioxidant stress, anti-inflammation, lipid metabolism regulation, and autophagy activation), but by an integrated regulatory network with tight multi-level crosstalk between each functional module, which is strongly supported by our pooled findings that salidroside synchronously reversed aberrant levels of hepatic lipid deposition, liver function impairment, oxidative stress, inflammatory response, and insulin resistance in NAFLD rodents. Specifically, oxidative stress and inflammatory response form a bidirectional vicious cycle as the core driver of NAFLD progression: CYP2E1/Nox-mediated excessive reactive oxygen species (ROS) production not only causes direct lipid peroxidation damage to hepatocytes, but also acts as a key molecular bridge to activate the NF-κB/MAPK signaling pathway and TXNIP-NLRP3 inflammasome, triggering the transcriptional release of pro-inflammatory cytokines including IL-6, IL-1β and MCP-1; in turn, the activated inflammatory cascade further upregulates ROS-generating enzymes and impairs the endogenous antioxidant system (reducing SOD and GSH activity), amplifying hepatic oxidative damage in a positive feedback loop, and salidroside simultaneously targets multiple key nodes of this cycle to block the progressive amplification of liver injury. Meanwhile, lipid metabolism disorder is the initiating link of the entire NAFLD pathological cascade, and salidroside exerts a combined lipid-lowering and lipotoxicity-clearing effect via dual regulation: on one hand, it directly reprograms hepatic lipid homeostasis through the AMPK/PPARα axis, accelerating fatty acid β-oxidation via upregulating CPT1α and LPL while inhibiting *de novo* lipogenesis via suppressing FAS and SREBP-1c, thus reducing intrahepatic triglyceride and cholesterol deposition and the resulting lipotoxicity at the source; on the other hand, it activates complete autophagic flux to promote the formation of lipophagosomes that specifically encapsulate and degrade excess lipid droplets in hepatocytes, which not only directly alleviates hepatic steatosis in combination with the lipid metabolism pathway, but also eliminates lipotoxicity-induced mitochondrial dysfunction and ROS overproduction, thus indirectly inhibiting the downstream oxidative stress and inflammatory cascades. Notably, autophagy acts as the central hub of the entire regulatory network: it not only bridges and interacts with the lipid metabolism pathway to reduce steatosis, but also alleviates oxidative stress and inflammatory response by clearing damaged organelles and misfolded proteins, while the improved intrahepatic redox and inflammatory microenvironment further reverses the impairment of autophagic flux caused by excessive ROS and pro-inflammatory factors, forming a benign positive feedback regulation.

In addition, at the mechanistic level, apart from the aforementioned four pathways, salidroside may also exert a therapeutic effect on NAFLD by regulating gut microbiota and improving insulin resistance. Regarding the regulation of gut microbiota, salidroside can modulate the composition of gut microbiota—such as enriching beneficial bacterial flora and inhibiting the excessive proliferation of pathogenic bacteria—to maintain the integrity of the intestinal barrier, thereby optimizing the regulatory function of the gut-liver axis and alleviating liver tissue damage. Existing studies have confirmed that gut microbiota modulation strategies, such as fecal microbiota transplantation and probiotic intervention, exhibit significant therapeutic effects on MAFLD (Liu et al., 2023; Shi et al., 2025). In terms of improving insulin resistance, the PI3K-AKT signaling pathway is a key pathway regulating hepatic insulin sensitivity. Salidroside can activate this pathway to promote GLUT4-mediated glucose transport and uptake, while inhibiting the activity of key gluconeogenic enzymes such as PEPCK and G6Pase. Consequently, it improves hepatic insulin resistance and alleviates dyslipidemia (Liu et al., 2025; Tang et al., 2025).

Salidroside is a phenylethanoid glycoside with antioxidant properties. It has been reported that certain small molecules with redox activity may act as pan-assay interference compounds (PAINS) (Bolz et al., 2021), leading to non-specific assay signals *in vitro*. Notably, Zhang et al. (2021) comprehensively summarized the pharmacological properties of salidroside and explicitly stated that its chemical structure (phenylethanoid glycoside, molecular formula C_14_H_20_O_7_) lacks the core structural motifs of typical PAINS compounds (e.g., Michael acceptors, redox cyclers, heavy metal chelators, or fluorescent chromophores). This conclusion is consistent with the structural characteristics described in Bolz et al. (2021), further supporting that salidroside does not exhibit PAINS-related non-specific assay interference. Importantly, the present meta-analysis exclusively included *in vivo* rodent studies rather than isolated enzyme-based *in vitro* assays. Therefore, the pooled therapeutic effects reported here are derived from whole-animal models, which reduces the likelihood that the main efficacy outcomes (hepatic TG, NAS score, ALT/AST) are merely assay artefacts. However, we acknowledge that many mechanistic conclusions in the included studies are based on signaling pathway modulation (e.g., Nrf2, AMPK, NLRP3) assessed through protein expression analysis rather than direct target-binding validation. Therefore, it cannot be excluded that some reported multi-pathway effects reflect secondary downstream consequences of general antioxidative activity rather than highly specific molecular interactions. At present, the evidence supporting salidroside for NAFLD is still in the preclinical stage, and there is a lack of high-quality randomized controlled clinical trial data. Future studies should incorporate orthogonal validation strategies, including direct target engagement assays, genetic loss-of-function models, and clinical investigation, to strengthen mechanistic credibility and translational potential.

In our meta-analysis, the existing outcome indicators have been described in detail; however, there are still limitations due to potential factors such as quality assessment and publication bias. These limitations include but are not limited to: the effect of different feeding methods on salidroside treatment; the intervention of regional differences on the experimental results, etc. We require additional high-quality studies to enhance our analysis and further validate the specific efficacy and safety of our findings.

Furthermore, our subgroup analysis revealed significant dose- and time-dependent therapeutic characteristics of salidroside in rodent models of non-alcoholic fatty liver disease (NAFLD), wherein doses ≥100 mg/kg/d yielded superior improvements in hepatic triglyceride and serum total cholesterol, while a treatment duration of ≤4 weeks achieved more pronounced reductions in serum alanine transaminase and aspartate transaminase, with these phenomena underpinned by well-defined molecular mechanisms closely tied to the pharmacological properties of salidroside, the activation characteristics of its core therapeutic targets, and the pathological progression features of NAFLD. For the superior lipid-lowering efficacy of high-dose salidroside, the core molecular basis lies in the dose-dependent activation pattern of its key targets peroxisome proliferator-activated receptor α (PPARα) and AMP-activated protein kinase (AMPK): doses ≥100 mg/kg/d can reach the hepatic activation saturation threshold of these two targets to fully drive fatty acid β-oxidation and inhibit *de novo* lipogenesis, while overcoming the first-pass metabolic limitation to maintain effective hepatic drug exposure, simultaneously activating the regulatory network encompassing antioxidant stress, anti-inflammation, lipid metabolism regulation and autophagy activation, and counteracting the persistent lipotoxicity in high-fat diet-induced NAFLD models, ultimately exerting a more robust lipid-modulating effect. For the more significant hepatoprotective effect of short-term treatment, the underlying mechanism is mainly attributed to the differential response of NAFLD-related liver injury to salidroside intervention: within the first 4 weeks of modeling, elevated liver enzymes are mainly derived from reversible acute hepatocyte damage caused by lipotoxicity, oxidative stress and transient inflammatory response, which can be rapidly alleviated by the antioxidant, anti-inflammatory and hepatocyte membrane-protective effects of salidroside; in contrast, prolonged modeling and treatment (>4 weeks) lead to chronic progressive liver injury accompanied by early fibrotic changes that are difficult to reverse, alongside adaptive desensitization of core therapeutic targets and upregulation of hepatic drug-metabolizing enzymes that accelerate salidroside clearance, thus resulting in a relatively reduced improvement amplitude of liver function indicators, despite the stable regulatory effect of long-term salidroside intervention on systemic metabolism and hepatic lipid deposition.

In addition, there were significant publication bias in the main outcome indicators and secondary outcome indicators in the article, which is a common problem in preclinical animal experiment meta-analyses. The main potential causes are as follows: (1) Negative results of animal experiments are less likely to be published in peer-reviewed journals; (2) Research groups are more inclined to report positive results of drug efficacy, leading to a lack of negative/neutral result studies in the included literature; (3) Most of the included studies are small-sample preclinical experiments, which are more likely to produce positive result bias due to small sample size and high random error. The publication bias in this study may lead to a slight overestimation of the efficacy of salidroside, especially for liver function indicators (ALT/AST) and inflammatory response indicators with the most significant publication bias. However, the sensitivity analysis showed that the pooled effect sizes of the two core primary endpoints (hepatic TG and NAS) had no significant changes after excluding any single study, indicating that the core conclusion of salidroside improving hepatic lipid deposition and pathological severity of NAFLD was not fundamentally affected by publication bias. To reduce the impact of publication bias on preclinical research conclusions, future studies should focus on large-sample, multi-center animal experiments, and the research results (including negative/neutral results) should be published in accordance with the pre-registration protocol (this study has been pre-registered in PROSPERO).

It is worth noting, however, that there are significant species differences between rodents and humans. This makes it difficult for existing animal models to simulate the complex pathological characteristics of NAFLD in humans, which poses a challenge to the effectiveness of translating preclinical research conclusions into clinical practice. In future studies, we need more high-quality clinical studies to verify its safety and efficacy.

## Data Availability

The original contributions presented in the study are included in the article/Supplementary Material, further inquiries can be directed to the corresponding author.

## References

[B1] Almohawes Z. N. El-Kott A. Morsy K. Shati A. A. El-Kenawy A. E. Khalifa H. S. (2022). Salidroside inhibits insulin resistance and hepatic steatosis by downregulating miR-21 and subsequent activation of AMPK and upregulation of PPARα in the liver and muscles of high fat diet-fed rats. Arch. Physiol. Biochem. 130 (3), 257–274. 10.1080/13813455.2021.2024578 35061559

[B2] Bolz S. N. Adasme M. F. Schroeder M. (2021). Toward an understanding of pan-assay interference compounds and promiscuity: a structural perspective on binding modes. J. Chem. Inf. Model 61 (5), 2248–2262. 10.1021/acs.jcim.0c01227 33899463

[B3] Chang L. Karin M. (2001). Mammalian MAP kinase signalling cascades. Nature 410 (6824), 37–40. 10.1038/35065000 11242034

[B4] Chu X. Liu S. Qu B. Xin Y. Lu L. (2024). Salidroside may target PPARα to exert preventive and therapeutic activities on NASH. Front. Pharmacol. 15, 1433076. 10.3389/fphar.2024.1433076 39415834 PMC11479876

[B5] Feng Q. Liu C. Gao W. Geng X. L. Dai N. (2019). Salidroside-Mitigated inflammatory injury of hepatocytes with non-alcoholic fatty liver disease *via* inhibition TRPM2 ion channel activation. Diabetes Metab. Syndr. Obes. 12, 2755–2763. 10.2147/dmso.S210764 31920355 PMC6938192

[B6] Gordy C. He Y. W. (2012). The crosstalk between autophagy and apoptosis: where does this lead? Protein Cell 3 (1), 17–27. 10.1007/s13238-011-1127-x 22314807 PMC4875212

[B7] Henao-Mejia J. Elinav E. Jin C. Hao L. Mehal W. Z. Strowig T. (2012). Inflammasome-mediated dysbiosis regulates progression of NAFLD and obesity. Nature 482 (7384), 179–185. 10.1038/nature10809 22297845 PMC3276682

[B8] Higgins J. P. Thompson S. G. Deeks J. J. Altman D. G. (2003). Measuring inconsistency in meta-analyses. Bmj 327 (7414), 557–560. 10.1136/bmj.327.7414.557 12958120 PMC192859

[B9] Ho S. L. Lin C. T. Lee S. S. (2021). *In silico* design and synthesis of N-arylalkanyl 2-naphthamides as a new class of non-purine xanthine oxidase inhibitors. Drug Dev. Res. 82 (6), 789–801. 10.1002/ddr.21782 33398913

[B10] Hong-shan L. I. Shao-dong CHEN De-zhou L. (2017). Intervention effect of salidroside on liver fat synthesis and oxidation of non-alcoholic fatty liver in rats induced by high-fat diet. China J. Traditional Chin. Med. Pharm. 32 (10), 4625–4628.

[B11] Hooijmans C. R. Rovers M. M. de Vries R. B. Leenaars M. Ritskes-Hoitinga M. Langendam M. W. (2014). SYRCLE's risk of bias tool for animal studies. BMC Med. Res. Methodol. 14, 43. 10.1186/1471-2288-14-43 24667063 PMC4230647

[B12] Hu M. Zhang D. Xu H. Zhang Y. Shi H. Huang X. (2021). Salidroside activates the AMP-Activated protein kinase pathway to suppress nonalcoholic steatohepatitis in mice. Hepatology 74 (6), 3056–3073. 10.1002/hep.32066 34292604

[B13] Iliopoulos D. Drosatos K. Hiyama Y. Goldberg I. J. Zannis V. I. (2010). MicroRNA-370 controls the expression of microRNA-122 and Cpt1alpha and affects lipid metabolism. J. Lipid Res. 51 (6), 1513–1523. 10.1194/jlr.M004812 20124555 PMC3035515

[B14] Jahn D. Kircher S. Hermanns H. M. Geier A. (2019). Animal models of NAFLD from a hepatologist's point of view. Biochim. Biophys. Acta Mol. Basis Dis. 1865 (5), 943–953. 10.1016/j.bbadis.2018.06.023 29990551

[B15] Jun Z. Zheyun H. E. Jing Z. Zhanyang X. I. A. Hongshan L. I. (2024). The mechanism of effect of Salidroside modulating gut flora intreating nonalcoholic fatty liver disease. Chin. J. Microecology 36 (8), 876–885. 10.13381/j.cnki.cjm.202408002

[B16] Lee G. H. Bhandary B. Lee E. M. Park J. K. Jeong K. S. Kim I. K. (2011). The roles of ER stress and P450 2E1 in CCl(4)-induced steatosis. Int. J. Biochem. Cell Biol. 43 (10), 1469–1482. 10.1016/j.biocel.2011.06.010 21722752

[B17] Li H. S. (2018). Salidroside and curcumin formula prevents liver injury in Nonalcoholic Fatty liver disease in rats. Ann. Hepatol. 17 (5), 769–778. 10.5604/01.3001.0012.3135 30145577

[B18] Li H. Ying H. Hu A. Li D. Hu Y. (2017). Salidroside modulates insulin signaling in a rat model of nonalcoholic steatohepatitis. Evid. Based Complement. Altern. Med. 2017, 9651371. 10.1155/2017/9651371 28255329 PMC5309415

[B19] Li H. Xi Y. Xin X. Tian H. Hu Y. (2020). Salidroside improves high-fat diet-induced non-alcoholic steatohepatitis by regulating the gut microbiota-bile acid-farnesoid X receptor axis. Biomed. Pharmacother. 124, 109915. 10.1016/j.biopha.2020.109915 31986416

[B20] Liang K. Ma S. Luo K. Wang R. Xiao C. Zhang X. (2024). Salidroside: an overview of its promising potential and diverse applications. Pharm. (Basel) 17 (12), 1703. 10.3390/ph17121703 39770545 PMC11678419

[B21] Liu J. Ding M. Bai J. Luo R. Liu R. Qu J. (2023). Decoding the role of immune T cells: a new territory for improvement of metabolic-associated fatty liver disease. Imeta 2 (1), e76. 10.1002/imt2.76 38868343 PMC10989916

[B22] Liu Z.-H. Yu B.-B. Zhou H.-H. Zhang S.-S. Yang X. Chen Z.-F. (2025). Characteristic components from Rehmannia radix and their effects on insulin resistance through PI-3K/AKT signaling pathway in HepG2 Cells. Food and Med. Homol. 2 (4), 9420073. 10.26599/FMH.2025.9420073

[B23] Ning D. Zou Y. Hui-fang WANG Mu-gen D. (2013). Inhibitory effect of salidroside on liver oxidative stress in rats with non-alcoholic steatohepatitis. Chin. J. Pathophysiol. 29 (9), 1704–1708. 10.3969/j.issn.1000-4718.2013.09.030

[B24] Pahl H. L. (1999). Activators and target genes of Rel/NF-kappaB transcription factors. Oncogene 18 (49), 6853–6866. 10.1038/sj.onc.1203239 10602461

[B25] Qu B. Liu X. Liang Y. Zheng K. Zhang C. Lu L. (2022). Salidroside in the treatment of NAFLD/NASH. Chem. Biodivers. 19 (12), e202200401. 10.1002/cbdv.202200401 36210339

[B26] Riazi K. Azhari H. Charette J. H. Underwood F. E. King J. A. Afshar E. E. (2022). The prevalence and incidence of NAFLD worldwide: a systematic review and meta-analysis. Lancet Gastroenterol. Hepatol. 7 (9), 851–861. 10.1016/s2468-1253(22)00165-0 35798021

[B27] Rong L. Zou J. Ran W. Qi X. Chen Y. Cui H. (2022). Advancements in the treatment of non-alcoholic fatty liver disease (NAFLD). Front. Endocrinol. (Lausanne) 13, 1087260. 10.3389/fendo.2022.1087260 36726464 PMC9884828

[B28] Rongjun L. I. Chunxia X. U. Zhang T. Zhang Z. Lv W. (2024). Role of salidroside in a mouse model of non-alcoholic fatty liver disease: a study based on nicotinamide phosphoribosyltransferase. J. Clin. Hepatology 40 (01), 64–69.

[B29] Shah A. Kumar S. Simon S. D. Singh D. P. Kumar A. (2013). HIV gp120-and methamphetamine-mediated oxidative stress induces astrocyte apoptosis *via* cytochrome P450 2E1. Cell Death Dis. 4 (10), e850. 10.1038/cddis.2013.374 24113184 PMC3824683

[B30] Shao-dong CHEN Hai-hong ZHOU Zheng-xiao ZHAO Jin L. I. U. Yu-mei ZHANG Guo-hui L. (2013). Jiangzhi and Hepatoprotective effect of salidroside on nonalcoholic fatty liver disease. China J. Traditional Chin. Med. Pharm. 28 (9), 2701–2703.

[B31] Shi Y. Chen Z. Fang T. Chen X. Deng Y. Qin H. (2025). Gut microbiota in treating inflammatory digestive diseases: current challenges and therapeutic opportunities. Imeta 4 (1), e265. 10.1002/imt2.265 40027479 PMC11865350

[B32] Sun A. Q. Ju X. L. (2021). Advances in research on anticancer properties of salidroside. Chin. J. Integr. Med. 27 (2), 153–160. 10.1007/s11655-020-3190-8 32144560

[B33] Tang Q. Liang Z.-H. Yuan J.-J. Shaaban E. H. H. Wei J.-F. Liu Z.-H. (2025). FER1L6 ameliorates insulin resistance by regulating GLUT4 expression. Food and Med. Homol. 2 (4), 9420070. 10.26599/FMH.2025.9420070

[B34] Vujkovic M. Ramdas S. Lorenz K. M. Guo X. Darlay R. Cordell H. J. (2022). A multiancestry genome-wide association study of unexplained chronic ALT elevation as a proxy for nonalcoholic fatty liver disease with histological and radiological validation. Nat. Genet. 54 (6), 761–771. 10.1038/s41588-022-01078-z 35654975 PMC10024253

[B35] Wang Z. Liu H. Li L. Li Y. Yan H. Yuan Y. (2022). Modulation of disordered bile acid homeostasis and hepatic tight junctions using salidroside against hepatocyte apoptosis in Furan-Induced mice. J. Agric. Food Chem. 70 (32), 10031–10043. 10.1021/acs.jafc.2c04654 35939816

[B36] Wu Y. L. Lian L. H. Jiang Y. Z. Nan J. X. (2009). Hepatoprotective effects of salidroside on fulminant hepatic failure induced by D-galactosamine and lipopolysaccharide in mice. J. Pharm. Pharmacol. 61 (10), 1375–1382. 10.1211/jpp/61.10.0015 19814871

[B37] Xue R. Yang R. X. Fan J. G. (2022). Epidemiological trends and clinical characteristic of NAFLD/MAFLD in Asia. J. Dig. Dis. 23 (7), 354–357. 10.1111/1751-2980.13117 35880548

[B38] Yang Z. R. Wang H. F. Zuo T. C. Guan L. L. Dai N. (2016). Salidroside alleviates oxidative stress in the liver with non-alcoholic steatohepatitis in rats. BMC Pharmacol. Toxicol. 17, 16. 10.1186/s40360-016-0059-8 27075663 PMC4831194

[B39] Ye S. S. Zeng Y. Y. Yin L. L. (2011). Effects of salidroside on proliferation, apoptosis, phagocytosis, ROS and NO production of murine peritoneal macrophages *in vitro* . Xi Bao Yu Fen Zi Mian Yi Xue Za Zhi 27 (3), 237–241. 21419037

[B40] Zhang X. Kuang G. Wan J. Jiang R. Ma L. Gong X. (2020). Salidroside protects mice against CCl4-induced acute liver injury *via* down-regulating CYP2E1 expression and inhibiting NLRP3 inflammasome activation. Int. Immunopharmacol. 85, 106662. 10.1016/j.intimp.2020.106662 32544869

[B41] Zhang X. Xie L. Long J. Xie Q. Zheng Y. Liu K. (2021). Salidroside: a review of its recent advances in synthetic pathways and pharmacological properties. Chem. Biol. Interact. 339, 109268. 10.1016/j.cbi.2020.109268 33617801

[B42] Zhang J. Zhou J. He Z. Xia Z. Liu H. Wu Y. (2025). Salidroside attenuates NASH through regulating bile acid-FXR/TGR5 signaling pathway *via* targeting gut microbiota. Int. J. Biol. Macromol. 307 (Pt 4), 142276. 10.1016/j.ijbiomac.2025.142276 40118401

[B43] Zheng T. Yang X. Li W. Wang Q. Chen L. Wu D. (2018). Salidroside Attenuates high-fat diet-induced nonalcoholic Fatty liver disease *via* AMPK-Dependent TXNIP/NLRP3 pathway. Oxid. Med. Cell Longev. 2018, 8597897. 10.1155/2018/8597897 30140371 PMC6081551

[B44] Zhou R. Tardivel A. Thorens B. Choi I. Tschopp J. (2010). Thioredoxin-interacting protein links oxidative stress to inflammasome activation. Nat. Immunol. 11 (2), 136–140. 10.1038/ni.1831 20023662

[B45] Zhu M. Yu J. (2024). Salidroside alleviates ferroptosis in FAC-induced Age-related macular degeneration models by activating Nrf2/SLC7A11/GPX4 axis. Int. Immunopharmacol. 142 (Pt A), 113041. 10.1016/j.intimp.2024.113041 39260309

[B46] Zhuang W. Yue L. Dang X. Chen F. Gong Y. Lin X. (2019). Rosenroot (Rhodiola): potential applications in aging-related diseases. Aging Dis. 10 (1), 134–146. 10.14336/ad.2018.0511 30705774 PMC6345333

