# Supplementary material for: Protective effects of salidroside on NAFLD rodent models by alleviating oxidative stress and inflammation: a meta-analysis and mechanism exploration
Source: Front Pharmacol. 2026 Apr 8;17:1709953. doi: 10.3389/fphar.2026.1709953 (PMC13099308; doi:10.3389/fphar.2026.1709953)
Supplement: Supplementary file 1 [file Table1.docx]

Supplementary Material

# Supplementary Tables 1

| NAFLD | Nonalcoholic Fatty Liver Disease |
| --- | --- |
| NASH | Nonalcoholic Steatohepatitis |
| NAS | NAFLD Activity Score |
| DAMPs | Damage-Associated Molecular Patterns |
| PAMPs | Pathogen-Associated Molecular Patterns |
| NLRs | NOD-Like Receptors |
| NLRP3 | NOD-Like Receptor Pyrin Domain-Containing Protein 3 |
| ALT | Alanine Transaminase |
| AST | Aspartate Transaminase |
| TG | Triglycerides |
| TC | Total Cholesterol |
| LDL-C | Low-Density Lipoprotein Cholesterol |
| HDL-C | High-Density Lipoprotein Cholesterol |
| NEFA | Nonesterified Fatty Acid |
| FBG | Fasting Blood Glucose |
| FSI | Fasting Serum Insulin |
| HOMA-IR | Homeostasis Model Assessment-Insulin Resistance |
| ROS | Reactive Oxygen Species |
| MDA | Malondialdehyde |
| GSH | Glutathione |
| SOD | Superoxide Dismutase |
| GPX | Glutathione Peroxidase |
| CAT | Catalase |
| NAC | N-Acetylcysteine |
| IL-6 | Interleukin-6 |
| IL-1β | Interleukin-1β |
| IL-10 | Interleukin-10 |
| IL-18 | Interleukin-18 |
| TNF-α | Tumor Necrosis Factor-α |
| MCP-1/MCP1 | Monocyte Chemoattractant Protein 1 |
| HYP | Hydroxyproline |
| TUNEL | Terminal Deoxynucleotidyl Transferase dUTP Nick-End Labeling |
| NADPH | Nicotinamide Adenine Dinucleotide Phosphate (Reduced Form) |
| NADP⁺ | Nicotinamide Adenine Dinucleotide Phosphate (Oxidized Form) |
| CYP2E1 | Cytochrome P450 2E1 |
| AMPK | AMP-Activated Protein Kinase |
| PPARα | Peroxisome Proliferator-Activated Receptor α |
| Nrf2 | Nuclear Factor Erythroid 2-Related Factor 2 |
| NF-κB | Nuclear Factor κB |
| ACC/Acc1 | Acetyl-CoA Carboxylase / Acetyl-CoA Carboxylase 1 |
| FAS | Fatty Acid Synthase |
| DGAT2 | Diacylglycerol Acyltransferase 2 |
| SREBP-1c | Sterol Regulatory Element-Binding Protein-1c |
| LC3 | Microtubule-Associated Protein 1 Light Chain 3 |
| SQSTM1 (p62) | Sequestosome 1 |
| BCL-2 | B-Cell Lymphoma 2 |
| BAX | BCL-2-Associated X Protein |
| TGF-β1 | Transforming Growth Factor-β1 |
| TRX | Thioredoxin |
| TXNIP | Thioredoxin-Interacting Protein |
| Nox2/Nox4 | NADPH Oxidase 2 / NADPH Oxidase 4 |
| CPT1α | Carnitine Palmitoyltransferase 1α |
| LPL | Lipoprotein Lipase |
| SDS | Salidroside |
| PO | Palmitic Acid/Oleic Acid |
| CD4⁺T | CD4-Positive T Lymphocyte |
| CD8⁺T | CD8-Positive T Lymphocyte |
| Tregs | Regulatory T Cells |
| Th17 | T Helper 17 Cells |
| NK | Natural Killer Cells |
| miRNA | MicroRNA |
