# Supplementary material for: Protective effects of salidroside on NAFLD rodent models by alleviating oxidative stress and inflammation: a meta-analysis and mechanism exploration
Source: Front Pharmacol. 2026 Apr 8;17:1709953. doi: 10.3389/fphar.2026.1709953 (PMC13099308; doi:10.3389/fphar.2026.1709953)
Supplement: Supplementary file 2 [file DataSheet1.docx]

Supplementary Material

# Supplementary Data

**Retrieval Strategy**

Pubmed：

#1 (((rhodioloside[MeSH Terms]) OR (sallidroside[Title/Abstract])) OR (rhodosin[Title/Abstract])) OR (salidroside[Title/Abstract])

#2 ((((((Non-alcoholic Fatty Liver Disease[MeSH Terms]) OR (Non alcoholic Fatty Liver Disease[Title/Abstract])) OR (Nonalcoholic Fatty Liver[Title/Abstract])) OR (NAFLD[Title/Abstract])) OR (Nonalcoholic Fatty Liver Disease[Title/Abstract])) OR (Nonalcoholic Steatohepatitis[Title/Abstract])) OR (Nonalcoholic Steatohepatitides[Title/Abstract])

#3 #1 AND #2

Embase：

#1 'salidroside'/exp

#2 'rhodioloside':ti,ab,kw OR 'sallidroside':ti,ab,kw OR 'rhodosin':ti,ab,kw OR 'salidroside':ti,ab,kw

#3 #1 OR #2

#4 'nonalcoholic fatty liver'/exp

#5 'non alcoholic fatty liver disease':ti,ab,kw OR 'nonalcoholic fatty liver':ti,ab,kw OR 'nafld':ti,ab,kw OR 'nonalcoholic fatty liver disease':ti,ab,kw OR 'nonalcoholic steatohepatitis':ti,ab,kw OR 'nonalcoholic steatohepatitides':ti,ab,kw

#6 #4 OR #5

#7 #3 AND #6

Cochrane library：

#1 MeSH descriptor: [rhodioloside] explode all trees

#2 (rhodioloside):ti,ab,kw OR (sallidroside):ti,ab,kw OR (rhodosin):ti,ab,kw OR (salidroside)ti,ab,kw

#3 #1 OR #2

#4 MeSH descriptor: [Non-alcoholic Fatty Liver Disease] explode all trees

#5 (Non-alcoholic Fatty Liver Disease):ti,ab,kw OR (Non alcoholic Fatty Liver Disease):ti,ab,kw OR (Nonalcoholic Fatty Liver):ti,ab,kw OR (NAFLD):ti,ab,kw OR (Nonalcoholic Fatty Liver Disease):ti,ab,kw OR (Nonalcoholic Steatohepatitis):ti,ab,kw OR (Nonalcoholic Steatohepatitides):ti,ab,kw

#6 #4 OR #5

#7 #3 AND #6

Web of Science Core Collection：

#1 ALL=(“rhodioloside” OR “sallidroside” OR “rhodosin” OR “salidroside”)

#2 ALL=(“Non-alcoholic Fatty Liver Disease” OR “Non alcoholic Fatty Liver Disease” OR “Nonalcoholic Fatty Liver” OR “NAFLD” OR “Nonalcoholic Fatty Liver Disease” OR “Nonalcoholic Steatohepatitis” OR “Nonalcoholic Steatohepatitides”)

#4 #1 AND #2

知网：

检索以高级检索，主题为

”红景天苷 + '红景天苷(sal)' + 红景天苷含量 + 红景天苷提取 + 红景天苷注射液 + 红景天苷单体”AND”非酒精性脂肪性肝 + 非酒精性脂肪性肝病 + 非酒精性脂肪性肝炎 + 非酒精性脂肪性肝疾病”

万方：

检索以高级检索，点击期刊论文、学位论文，主题为”红景天苷”AND”非酒精性脂肪肝 OR 非酒精性脂肪性肝病 OR 非酒精性脂肪性肝炎”

维普：

检索以高级检索，主题为

“红景天苷+salidroside+红景天甙” AND”非酒精性脂肪肝+nafld+non alcoholic fatty liver+non-alcoholic fatty liver+non-alcoholic fatty liver disease+非酒性脂肪肝+非酒精性脂肪肝病+非乙醇性脂肪肝+非乙醇性脂肪性肝病+非酒精性脂肪性肝病+非酒精性肝病”

SinoMed (CBM)：

#1 "红景天苷"[常用字段:智能]

#2 "非酒精性脂肪肝"[常用字段:智能] OR "非酒精性脂肪性肝病"[常用字段:智能] OR "非酒精性脂肪性肝炎"[常用字段:智能] OR "非酒精性脂肪性肝疾病"[常用字段:智能]

#3 (#2) AND (#1)
